# Divided omental flap wrapping a multiple-branched graft replaced with an infected thoracic aortic aneurysm: A case report^✰^

**DOI:** 10.1016/j.jpra.2023.04.001

**Published:** 2023-04-14

**Authors:** Hideyoshi Sato, Kazuhiro Toriyama, Ryo Ogawa, Toshiyuki Yamada, Hisao Suda

**Affiliations:** a Department of Plastic and Reconstructive Surgery, Nagoya City University Graduate School of Medical Sciences and Medical School, 1-Kawasumi, Mizuho-cho, Mizuho-ku, Nagoya 467-8602, Japan; b Department of Gastroenterological Surgery, Nagoya City University Graduate School of Medical Sciences and Medical School, 1-Kawasumi, Mizuho-cho, Mizuho-ku, Nagoya 467-8602, Japan; c Department of Cardiovascular Surgery, Nagoya City University Graduate School of Medical Sciences and Medical School, 1-Kawasumi, Mizuho-cho, Mizuho-ku, Nagoya 467-8602, Japan

**Keywords:** Artificial vascular graft, Divided omental flap, Epiploic vessel, Infected thoracic aortic aneurysm, Multiplex graft

## Abstract

The omental flap is often used to fill the space around the artificial vascular graft as a network sheet to prevent artificial vascular infection. In this study, we report a case in which the omental flap was divided into three parts to fill the dead spaces around the multiple-branched graft, as well as to wrap the suture lines of the graft after graft replacement in a patient with an infected thoracic aorta.

An 88-year-old woman was admitted to the hospital with fever and impaired consciousness. Computer tomography revealed an aortic arch aneurysm with enlargement. After emergency stent–graft interpolation and antibiotic treatment, an infected thoracic aortic aneurysm was removed, and a multiple-branched graft replacement of the upper arch was performed. After harvesting an omental flap based on the right gastroepiploic vessels, the omental flap was divided into three on the basis of the epiploic vessels. The middle part of the omental flap was used to fill the space around the lesser curvature of the arch and the distal anastomotic site, the accessory part was used to fill the space between the ascending aorta and the superior caval vein, and the right part was used to wrap the three cervical branches, separately. Fifteen months after surgery, the patient had recovered enough to resume work without any signs of inflammation.

## Introduction

Infected thoracic aortic aneurysms are relatively rare and difficult to treat. The aneurysm is often replaced by an artificial vascular graft, which is covered by an omental or muscle flap to prevent graft infection wherever possible.^1^ The omental flap is often wrapped around the graft as a network sheet.^1^^,^^2^ We herein report a case in which the omental flap was split into three parts based on the epiploic vessels to fill the complicated dead spaces and wrap the graft suture lines after graft replacement in a patient with an infected thoracic aorta.

## Case presentation

An 88-year-old woman was admitted to the hospital with fever and impaired consciousness. Computer tomography revealed an aortic arch aneurysm with enlargement. *Escherichia coli* was cultured from the urine and blood, and ceftriaxone was administered. Emergency stent–graft interpolation was performed 1 week after hospitalization, and the infection subsided after treatment with piperacillin/tazobactam.

**Figure 1:**
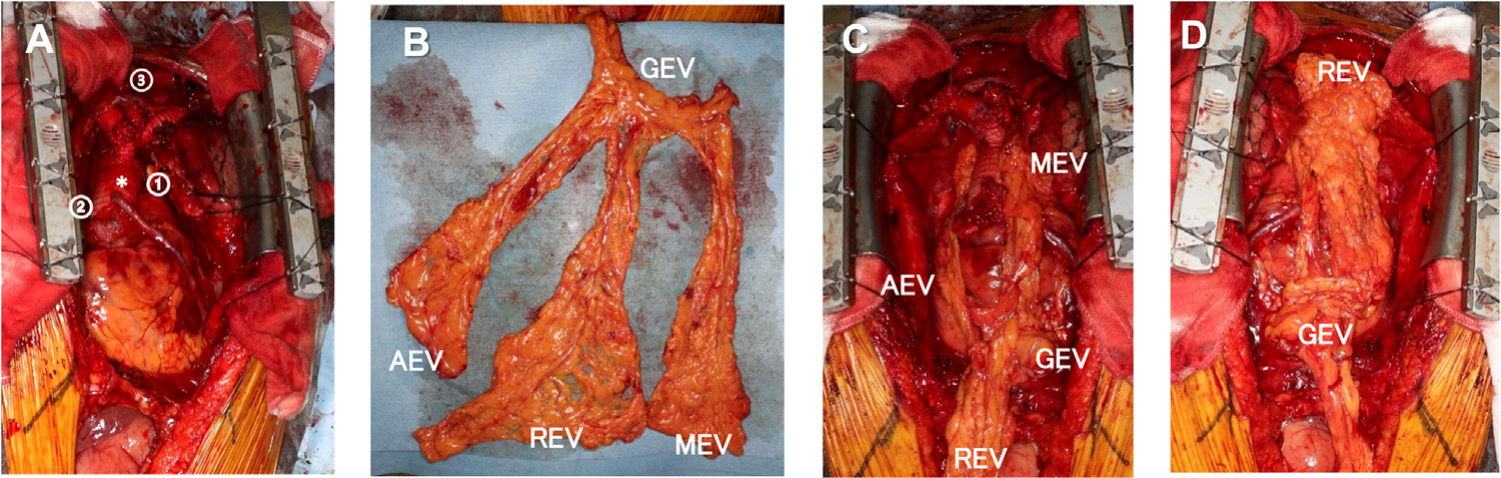
Filling and wrapping of the graft by dividing the omental flap into three parts. A: Multiplex graft (*) and transposition sites of the omental flap. (1) The space around the lesser curvature of the arch. (2) The space between the ascending aorta and the superior caval vein. (3) The suture lines at the three cervical branches. B: The divided omental flap. C: The MEV was used to fill the space in (1), and the AEV was used to fill the space in (2). D: The REV was used to wrap the graft suture lines in (3). GEV, gastroepiploic vessel; MEV, middle epiploic vessel; REV, right epiploic vessel; AEV, accessory epiploic vessel.

Five weeks after admission, the infected thoracic aortic aneurysm was removed, and a multiple-branched graft replacement of the upper arch was performed (Figure 1A). We harvested the omental flap based on the right gastroepiploic vessels. As long as the gastroepiploic vessels were preserved, the omental flap was divided into three on the basis of the middle, right, and accessory epiploic vessels (Figure 1B). The blood flow of the divided omental flap was confirmed with indocyanine green fluorescence imaging. The middle part was used to fill the space around the lesser curvature of the arch and the distal anastomotic site, the accessory part was used to fill the space between the ascending aorta and the superior caval vein (Figure 1C), and the right part was used to wrap the suture lines, especially at the three cervical branches (Figure 1D). The flap was secured with 4-0 absorbable monofilament sutures and covered the circumference of the graft. Within a few months after surgery, the patient experienced repeated pleural effusion and aspiration pneumonia. However, those symptoms had been improved with the 10-week application of antibiotics and the cardiac rehabilitation. 6 months after surgery, the patient had recovered enough to resume work without any signs of inflammation.

## Discussion

The omental flap is often used in patients with mediastinitis or infected aortic aneurysm. Moreover, it is often used to fill dead spaces as a network sheet without division. Settembre et al.^3^ demonstrated vascularization of the right gastroepiploic vessels, which supply several descending epiploic vessels. Zaha et al.^4^ divided the flap into two parts for the purpose of breast reconstruction. Moreover, Suito et al.^5^ divided the omental flap into three parts for the purpose of reconstructing a completely circumferentially degloved thumb. In this study, we divided the omental flap into three parts for the prevention of artificial graft infection.

Kim et al.^6^ emphasized that aggressive intraoperative debridement with soft-tissue coverage results in a high rate of success in high-risk patients with mycotic aortic aneurysm. Aggressive debridement may lead to complicated dead spaces around the multiple-branched graft, especially the lesser curvature of the arch and between the ascending aorta and the superior caval vein, which is where we filled the dead spaces by dividing the omental flap in the present case.

## Conclusion

The omental flap can be maximally utilized by confirming flap blood flow and dividing the flap into multiple parts as needed.

## Funding

None.

## Ethical approval

Not required.

## Declaration of Competing Interest

None.
